# Overexpression of the transcription factor Yap1 modifies intracellular redox conditions and enhances recombinant protein secretion

**DOI:** 10.15698/mic2014.11.173

**Published:** 2014-10-31

**Authors:** Marizela Delic, Alexandra B. Graf, Gunda Koellensperger, Christina Haberhauer-Troyer, Stephan Hann, Diethard Mattanovich, Brigitte Gasser

**Affiliations:** 1 Department of Biotechnology, BOKU University of Natural Resources and Life Sciences Vienna, Vienna, Austria.; 2 Austrian Centre of Industrial Biotechnology (ACIB), Vienna, Austria.; 3 School of Bioengineering, University of Applied Sciences FH Campus Wien, Vienna, Austria.; 4 Department of Chemistry, BOKU University of Natural Resources and Life Sciences Vienna, Vienna, Austria.

**Keywords:** ER, cytosol, cellular redox regulation, oxidative protein folding, glutathione, redox sensitive roGFP, Pichia pastoris

## Abstract

Oxidative folding of secretory proteins in the endoplasmic reticulum (ER) is a redox active process, which also impacts the redox conditions in the cytosol. As the transcription factor Yap1 is involved in the transcriptional response to oxidative stress, we investigate its role upon the production of secretory proteins, using the yeast *Pichia pastoris* as model, and report a novel important role of Yap1 during oxidative protein folding. Yap1 is needed for the detoxification of reactive oxygen species (ROS) caused by increased oxidative protein folding. Constitutive co-overexpression of *PpYAP1* leads to increased levels of secreted recombinant protein, while a lowered Yap1 function leads to accumulation of ROS and strong flocculation. Transcriptional analysis revealed that more than 150 genes were affected by overexpression of *YAP1*, in particular genes coding for antioxidant enzymes or involved in oxidation-reduction processes. By monitoring intracellular redox conditions within the cytosol and the ER using redox-sensitive roGFP1 variants, we could show that overexpression of *YAP1* restores cellular redox conditions of protein-secreting *P. pastoris* by reoxidizing the cytosolic redox state to the levels of the wild type. These alterations are also reflected by increased levels of oxidized intracellular glutathione (GSSG) in the *YAP1* co-overexpressing strain. Taken together, these data indicate a strong impact of intracellular redox balance on the secretion of (recombinant) proteins without affecting protein folding per se. Re-establishing suitable redox conditions by tuning the antioxidant capacity of the cell reduces metabolic load and cell stress caused by high oxidative protein folding load, thereby increasing the secretion capacity.

## INTRODUCTION

Cells have evolved potent antioxidant mechanisms in order to circumvent oxidative stress and oxidative damage of cellular components when reactive oxygen species (ROS) occur during physiological conditions. Apart from the mitochondrial respiratory chain and the beta-oxidation of fatty acids occurring in the peroxisomes, also oxidative protein folding of secretory proteins within the endoplasmic reticulum (ER) was reported to contribute to the formation of ROS (reviewed by 1,2,3,4).

Formation of disulfide bonds is attained through the oxidative protein folding machinery in the ER, using glutathione as redox buffer. In order to enable oxidative protein folding, the ER is specially equipped with dedicated enzymes such as oxidoreductases and chaperones and has a more oxidizing environment than the cytosol (for recent reviews see e.g. 5,6,7). During *de novo* formation of disulfide bonds, protein disulfide isomerase (PDI) introduces disulfide bonds into nascent client proteins, which causes reduction of PDI. FAD-dependent ER oxidoreductin Ero1 is responsible for the re-oxidation of PDI 8. Ero1 uses molecular oxygen as terminal electron acceptor, thereby generating stoichiometric amounts of hydrogen peroxide H_2_O_2_ 9,10. It has been suggested that glutathione acts as a redox buffer against ER-derived oxidative stress 11,12.

Once generated, ROS can damage nucleic acids and lead to oxidation of proteins and peroxidation of lipids 13. As a consequence, the expression of a set of proteins that eliminate ROS is induced 14, which is called "the oxidative stress response". Most organisms have evolved a combination of mechanisms for maintaining cellular redox balance, involving both ROS detoxifying enzymes with very high catalytic activity (such as superoxide dismutases (SODs), catalases and glutathione peroxidases), redox-regulating enzymes (e.g. thioredoxin, glutaredoxins, peroxiredoxins) as well as non-enzymatic compounds such as glutathione 3. The oxidative stress response is a complex regulatory circuit controlled by the interplay of different transcription factors, which are often subject to redox induced structural changes 4,15. Among them, the yeast AP-1 transcription factor Yap1 plays a major role in the regulation of the transcriptional response to oxidative stress 16.

Upon treatment of the yeast *Saccharomyces cerevisiae* with hydrogen peroxide, at least 115 genes are overexpressed and 52 repressed as a consequence of oxidative stress in the cell 17, the majority of which is regulated by Yap1p 18. Yap1p-mediated regulatory pathways are activated by redox-sensitive cysteine residues that serve as cellular sensors for changes in the intracellular redox balance (reviewed e.g. by 3 and 12). Through the formation of different intramolecular disulfide bonds, Yap1 undergoes different conformational changes upon exposure to different oxidants (such as hydrogen peroxide or the superoxide generating oxidant menadione), which result in nuclear localization of Yap1 by masking the leucine-rich nuclear export signal 19. Microarray data of a *YAP1* overexpressing *S. cerevisiae* strain revealed that oxidoreductases formed a remarkable fraction of the regulated genes. This group was supposed to have a protective function upon oxidative stress 20. Another important function of Yap1 and its target genes is to balance cytosolic redox homeostasis 21.

Using the yeast *Pichia pastoris* as a model, we have recently shown that protein folding stress within the ER has a strong impact on the redox state of the cytosol, leading to a significant reduction of this compartment 22. By applying redox sensitive variants of green fluorescent protein (roGFP) to measure *in vivo* glutathione redox conditions in ER and cytosol during oxidative protein folding, we observed a significant reduction of the redox state of the cytosol when ER resident proteins such as Ero1, Pdi1, or recombinant secretory proteins were overexpressed.

Cellular redox imbalance is stressful for the cells, and does not only lead to reduced productivity in biotechnological production of recombinant proteins, but is also associated with the development of many aging-related human diseases including diabetes mellitus, atherosclerosis, and neurodegenerative diseases such as Alzheimer’s, amyotrophic lateral sclerosis and Parkinson’s (reviewed e.g. by 23). Therefore we aimed at restoring cytosolic redox ratios during conditions of increased oxidative protein folding in the ER.

Yano *et al.* recently identified the *P. pastoris* Yap1 homolog and reported the involvement of the transcription factor in the detoxification of formaldehyde and ROS in cells grown on methanol as carbon and energy source 24,25. Here we investigated the role of Yap1 during the production of recombinant secretory proteins in glucose based growth conditions in *P. pastoris*, and report a novel role of Yap1 during ER resident oxidative protein folding.

## RESULTS AND DISCUSSION

### Downregulation of the transcription factor Yap1 leads to accumulation of reactive oxygen species (ROS), whereas Yap1 overproduction has a positive influence on protein secretion 

In our previous studies we reported that increased oxidative protein folding within the ER of *P. pastoris* led to changes in the redox state of the cytosol, independent of unfolded protein response (UPR) activation 22. Oxidative protein folding has been implicated with the generation of stoichiometric amounts of H_2_O_2_ during regeneration of reduced Pdi1 by Ero1 26, correspondingly, we detected ROS in strains with increased levels of Pdi1*. *However, we did not detect ROS accumulation upon increased folding load due to overexpression of secretory model proteins unless ER stress and UPR activation occurred 22. Therefore we assumed that the cellular ROS scavenging system is effective to degrade ROS under physiological levels of oxidative folding, but is overwhelmed upon severe ER stress, thus leading to ROS accumulation. As Yap1 could be involved in the detoxification of ROS generated during oxidative folding of proteins in the ER, we examined the role of this transcription factor during the production of the secretory model protein trypsinogen (TRP) in more detail. In order to observe the specific effects of oxidative protein folding, we used a TRP secreting strain which is not induced for UPR 22.

In this strain background (trp), we investigated the influence of lowered (trp∆*yap1*) and increased constitutive (trpYAP1) expression levels of *YAP1*. Increased constitutive expression of *YAP1* was obtained by integrating an additional copy of *YAP1* under the control of the strong glycolytic glyceraldehyde-3-phosphate dehydrogenase P*_GAP_* promoter into the *P. pastoris* genome 27, while down-regulation of the *YAP1 *expression level was achieved through the exchange of the native promoter of the respective gene with the serine repressible promoter P*_SER1_* 28. Repression of this promoter of the serine biosynthesis gene *SER1* was obtained through repeated addition of 10 mM serine to the synthetic M2 medium during the cultivation. We decided to go for a conditional *yap1* repression rather than a total gene knock out in order to be more flexible and prevent potential growth impairment as was reported for the *yap1* knock-out during growth on methanol 24,25. We did not analyse *YAP1* down-regulation in the wild type background, because so far no detectable phenotypic differences were reported for *yap1* mutants during normal non-stressed growth conditions (25 for *P. pastoris*, 29 for *S. cerevisiae*).

**Figure 1:**
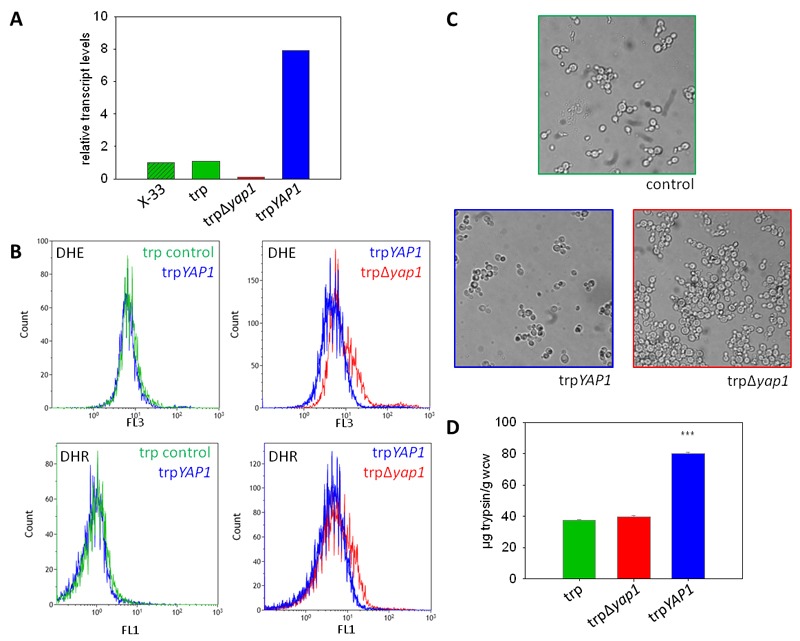
FIGURE 1: Downregulation of *YAP1* leads to accumulation of reactive oxygen species and flocculation, whereas *YAP1* co-overexpression enhances secretion of recombinant trypsinogen. **(A)** Transcript levels of *YAP1* measured by quantitative real time PCR in the strains X-33, trp, trp∆*yap1* and trpYAP1. **(B)** ROS were measured with the fluorescent dyes DHE and DHR in the wild type (green), the trpYAP1 strain (blue) and the trp∆*yap1* strain (red). **(C)** Microscopic images of X-33, trpYAP1 and trp∆*yap1 *after 20 h of cultivation in M2 medium. **(D)** Amounts of secreted trypsinogen in the strains trp, trp∆*yap1* and trpYAP1 measured with TAME assay. The average of 10 clones per strain and the standard error of the mean (SEM) are shown, the significance of differences is indicated by *** (P <0.01).

Transcript level determination with quantitative real time PCR showed clearly that the trp∆*yap1* strain expressed less than 10% of YAP1 mRNA compared to *P. pastoris* wild type X-33 or the parental trp strain (all grown in repressing conditions of P*_SER1_*), whereas the trpYAP1 strain exhibited significantly higher YAP1 mRNA levels (approx. 8-fold higher levels as compared to X-33 and the parental trp strain) (Figure 1A).

Next, we determined the levels of accumulated ROS in the Yap1 deregulated strains compared to their parental strain. DHR and DHE were used for detection of H_2_O_2_ and superoxide anion as described previously 22. As reported in 22, the non-UPR induced single copy trp strain did not accumulate significant levels of ROS compared to the wild type X-33 (data not shown). ROS analysis with flow cytometry revealed that trp∆*yap1* indeed accumulated significantly higher levels of both ROS species compared to the non-engineered control strain (Figure 1B), indicating its essential function in detoxification of accumulated ROS. Accumulation of ROS in the *YAP1 *overexpressing strain trpYAP1 was indistinguishable from the non-engineered single copy trp strain. Moreover, we observed a strong phenotype of the Yap1-depleted strain trp∆*yap1*, which showed intensive flocculation in liquid cultures (Figure 1C). As no phenotype has been reported upon *yap1 *deletion previously, we hypothesize that flocculation is a response triggered by the enhanced level of oxidative protein folding.

Oxidative stress response has been implicated with recombinant protein secretion stress in *S. cerevisiae* quite recently, using a transcriptomics based approach. Although Yap1 itself did not show up within the differentially regulated genes, the Reporter Transcription Factors algorithm 30 identified Yap1 as significant based on down-regulation of Yap1 target genes in a *S. cerevisiae* strain secreting α-amylase, a difficult to express recombinant protein 31. On the contrary, during our transcriptomics analyses of different recombinant protein secreting strains of *P. pastoris*, we did not see any significant regulation of putative Yap1 target genes involved in oxidative stress defense (32,33 and Stadlmayr et al. unpublished data). However, a significant downregulation of *YAP1*, and several of its target genes such as *AHP1*, *CTA1*, *GLR1*, *GRX3*, *GSH1*, *GSH2*, *SOD2*, and *TRX1* (among other genes) was observed in a *HAC1* overexpression *P. pastoris* strain (except for *SOD1* which was up-regulated; data from 34) correlating with ROS accumulation in this strain with constitutive UPR induction 22.

Analysis of trypsinogen secretion levels in the strains deregulated for Yap1 transcript levels substantiate a role of Yap1 in the secretion process. *YAP1* overexpression enhanced the level of secreted trypsinogen in the single copy trp strain more than 2-fold (Figure 1D). Notably, only the amount of correctly folded active protein is measured by the enzymatic TAME assay 35. No influence on biomass specific trypsinogen yields were observed in the conditional *yap1* knock-down strain (Figure 1D). Quantitative real time PCR analysis showed that transcript levels of genes involved in oxidative protein folding, such as *ERO1* and *PDI1*, which have been shown to enhance trypsinogen secretion previously 22, remained unchanged in the *YAP1* overexpressing strain (data not shown), ruling out one possible explanation for the enhanced secretion phenotype. Additionally, we confirmed the positive impact of *YAP1* overexpression on protein secretion also in a high level secreting and UPR-induced *P. pastoris* strain (containing multiple copies of the pTRP expression cassette), leading on average to 1.58-fold higher secretion yields.

### Genes involved in the antioxidant response are significantly up-regulated in the *YAP1*-overexpressing strain trpYAP1

In order to elucidate the cellular mechanisms behind the improved secretion of a recombinant protein through constitutive overexpression of *YAP1*, transcriptional analysis of this strain and its parental strain were performed during exponential growth of the cells using DNA microarrays 34. The *yap1* knock-down strain was excluded at this point, as its strong flocculation phenotype hampered quantitative sampling. This technical limitation was not an issue as our aim was to investigate the transcriptional regulation responsible for the increased secretion phenotype, and not to elucidate the Yap1 regulon upon oxidative stress in *P. pastoris*. Microarray analysis revealed that the constitutive overexpression of *YAP1* (without any external oxidative stimulus) exerted significant regulation of 170 genes (cutoff criteria: adjusted P-value < 0.05), 98 thereof being induced, and 72 being downregulated (Table 1). Out of these genes, 146 (more than 85%) had at least one YAP binding site (ARE) in their promoter regions (determined using Matinspector, Genomatix, see Additional File 1). Basal regulation through *YAP1* overexpression without stressors has also been reported for *S. cerevisiae* 20,36. Most probably, the transcriptional response without external stressor is due to overload of Crm1, the protein responsible for nuclear export of Yap1, and consequently increased levels of Yap1 localized to the nucleus (as suggested by 36). Localization was analysed using Yap1, which was C-terminally fused to sGFP and found to be distributed all over the cell including the nucleus (data not shown).

**Table 1:** Differentially regulated genes upon *YAP1* overexpression in *P. pastoris* secreting trypsinogen (trpYAP1/trp). Significantly up- and down-regulated genes were determined using an adjusted P-value <0.05 as cut-off criterion. Genes that exceed an expression fold change of ± 1.5 fold are highlighted in bold.

**Functional group**	**total**	**up**	**down**	**Gene names**
Amino acid metabolism,	10	2	8	⇑ *GLY1*,* HIS2*
thereof glutamine family	6	0	6	⇓ *ARG1*,*ARG5*,*6*,*CPA1*, ***GAD1***,* GCV2*, ***GLT1***,*HIS7*,* MET13*
Cell wall & glycosylation	14	4	10	⇑ ***PAS_chr048_0005***,* PAS_chr1-1_0135*, ***PAS_chr3_0030***, ***YJR061W***
				⇓ ***EXG1****, FLO103*,* FLO104*,* FLO11*, ***FLO5-2***,* GAS1-2*,* KTR1*,* PpBMT1*,* PpBMT2*,* UTR2*
Cofactor	4	1	3	⇑ *RIB3*
				⇓ *SNZ3*,* THI4*,* THI7*
Lipid metabolism	4	2	2	⇑ *PDR16*,* PRY2*
				⇓ ***INO1***,* RSB1*
Metabolism,	9	5	4	⇑ * ACS1*,*ArbD*,*GOR1*,*GPD1*, ***RGS2*** ⇓ *ALD5*,* DAL7*,* DOG1*, ***ICL1***
thereof oxido-reductase activity	4	3	1	⇓ *ALD5*,* DAL7*,* DOG1*, ***ICL1***
Metal ion homeostasis	20	11	9	⇑ *ATX1*, ***FET3***, ***FRE2***, ***FRE3***, ***FRE6***, ***FTR1***,* MZM1*, ***PAS_chr1-3_0013***, ***PAS_chr2-2_0470***, ***SIT1***,* YOR389W*
				⇓ ***CCC1***, ***CTR1***,* FEP1*,* FRA1-2*, ***FRE1***,* PAS_chr3_0141*, ***PHO84***,* PIC2*,* SMF2*
Other	8	6	2	⇑ *CWC15*,* ESS1*,* FCY1*,* IMP2*, ***PAS_chr1-4_0226***, ***PAS_chr4_0991***
				⇓ *CMK2*,* SLM1*
Response to oxidative stress	26	26	0	⇑ ***AHP1***, ***AIF1-1***,* CCS1*,* CYS3*,* ECM4*,* ETT1*, ***GSH1***,* HAP1*, ***HBN1***,* HYR1*,* MCR1*, ***OYE2***, ***OYE3***, ***PAS_c034_0013***,* PCD1*,* PST2*,* PTC7*, ***SNQ2***, ***SOD1***, ***SRX1***,* TRR1*, ***TRX1***, ***TRX2***, ***TSA1***, ***YAP1***, ***YDL124***
Oxido-reductase activity	11	10	1	⇑ ***ETR1***, ***FDH1***, ***PAS_c157_0001***, ***PAS_chr3_0006***, ***YDR541C-2***, ***YDR541C-3***, ***YDR541C-4***, ***YDR541C-5***, ***YNL134C-1***, ***YNL134C-3***
				⇓ ***PAS_chr2-1_0307***
Proteolysis,	5	4	1	⇑ *ADD66*,*PRE4*,*UBI4*,* YPS7*
thereof proteasome				⇓ *YPS1-4*
Transcriptional regulator,	10	7	3	⇑ *ACA1*, ***MPP1***, ***PAS_chr1-3_0166***,* PAS_chr1-4_0652*, ***TOS8***,* YPR013C*,* YPR022C-1*
also metal-ion homeostasis				⇓ *FEP1*,*FRA1-2*, ***PAS_chr4_0324***, ***RME1***,* YLR278C*
Transport,	18	6	12	⇑ *ATO2*, ***FLR1***,* PAS_chr1-4_0636*, ***SNG1***, ***TPO1***, ***YOR1-1***
thereof polyamine transport				⇓*AQR1*,* AQY1*, ***GAP1***,* ITR1*,* ITR2*,* MEP1*,* PAS_chr4_0656*, ***PDR5-2***,* PDR12*, ***TPO3***,*TPO4*,* YHL008C*
Unknown	31	14	17	⇑ ***BSC5***, ***ECM13***,* PAS_chr1-1_0094*,* PAS_chr1-1_0209*,* PAS_chr3_0153*,* PAS_chr3_0187*,* PAS_chr3_0288*, ***PAS_chr3_0837***, ***PAS_chr4_0080***,* PAS_chr4_0328*,* PAS_chr4_0773*, ***PAS_chr4_0820***,* PAS_chr4_0860*, ***YPR127W***
				⇓ *AIM17*,* APD1*,* DCG1*, ***PAS_chr1-1_0257***,* PAS_chr1-3_0169*, ***PAS_chr1-4_0689***, ***PAS_chr2-1_0064***,* PAS_chr2-1_0240*,* PAS_chr2-1_0539*, ***PAS_chr2-1_0642***,* PAS_chr3_0494*,* PAS_chr4_0602*, ***PAS_chr4_0851***,* PAS_chr4_0947*, ***TMA10***,* YBR056W*,* YMR244W*

GO Term Finder (P-value cutoff <0.02) was used to identify significantly regulated biological processes, molecular functions and cellular components (Table 1 and Additional File 1). Among the upregulated genes, GO processes “Response to oxidative stress”, “Response to chemical stimulus”, “Metal ion transport” and “Cellular homeostasis” were the most significantly influenced biological functions, while “glutamine family amino acid metabolism”, “manganese ion transport” and “flocculation” are significantly down-regulated functions.

Regulation of genes coding for multidrug transporter has been reported to be dependent on Yap1 37,38,39, but has not been shown without externally applied stress previously. Transcriptional regulation of genes involved in cellular iron and copper homeostasis might look contradictory at first, as there is no difference in the external ion concentrations, but are clearly related to the lowered levels of the GATA type repressor of the iron regulon Fep1 40 and the repressor Fra1-2 in the *YAP1* overexpressing strain. Seemingly, tight control of the uptake of these free redox active metal ions is a part of the antioxidant response. The down-regulation of the several cell wall associated genes including *FLO103*, *FLO104*, FLO11, *FLO5-2* upon *YAP1* overexpression may explain the strong flocculation phenotype of the *yap1* knock-down strain. Down-regulation of the flocculin genes might be due to the slight up-regulation of the gene encoding Nrg1 transcriptional repressor, which seems to be a key regulator of flocculation-related genes in *P. pastoris* (own unpublished results).

Contrary to previous regulation patterns observed in *S. cerevisiae* upon overexpression of Yap1 or oxidative stimulus 17,20, we do not see chaperones or other folding related genes among the regulated genes in our analysis, thus ruling out their impact on the improved secretion phenotype.

Among the regulated genes, increased expression of genes coding for antioxidant enzymes, e.g., superoxide dismutase and peroxiredoxins, was predominant (Table 1). Strong up-regulation of the majority of genes involved in cytosolic ROS detoxification such as superoxide dismutase *SOD1*, a second cytosolic Cu/Zn SOD isoenzyme encoded by PAS_c034_0013, SOD co-chaperone *CCS1*, peroxiredoxin *AHP1*, sulfoxiredoxin *SRX1* which is needed to reduce thioredoxin peroxidase encoded by *TSA1*, cytoplasmic thioredoxin peroxidase *TRR1* which keeps the thioredoxin system (*TRX1*, *TRX2*) in the reduced state as well as glutathione biosynthesis enzymes gamma glutamylcysteine synthetase *GSH1, *cystathionine gamma-lyase *CYS3* and omega glutathione transferase *ECM4* was observed, indicating that they might be correlated to enhanced secretion.

### Comparison of total intra- and extracellular glutathione (GSH) and glutathione disulfide (GSSG) levels show no significant differences, but an alteration in the GSH/GSSG ratio 

**Figure 2:**
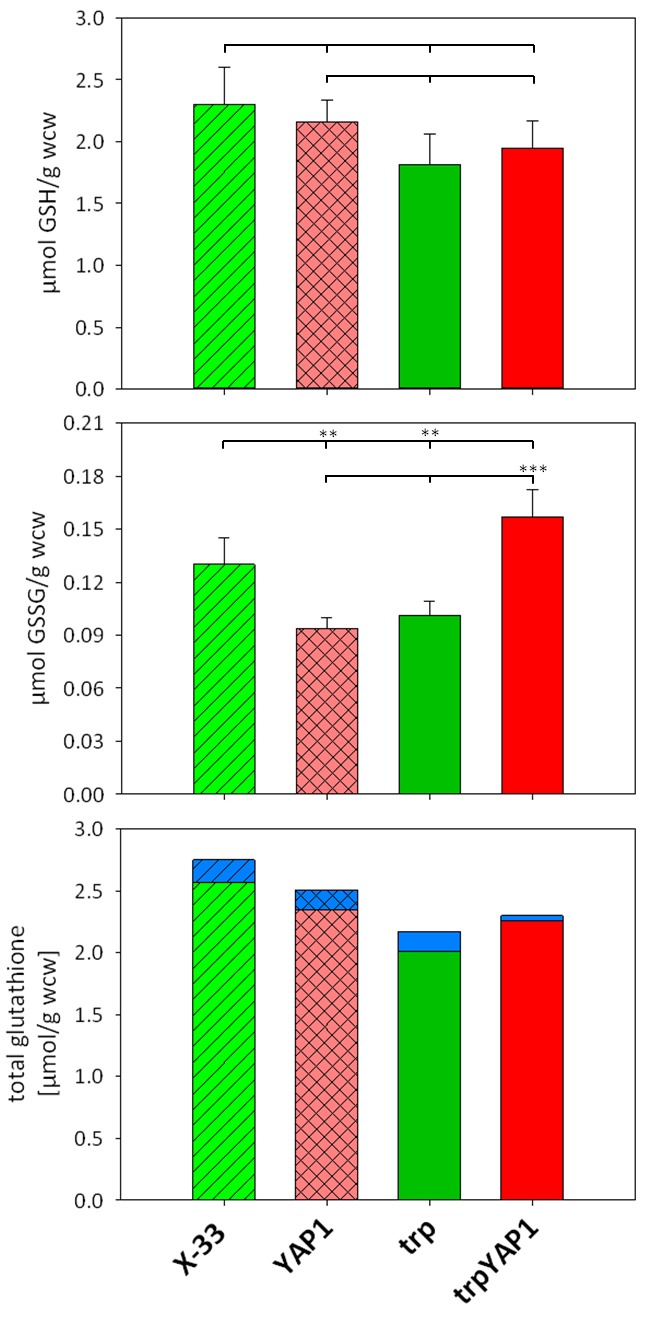
FIGURE 2: Comparison of intra- and extracellular glutathione (GSH) and glutathione disulfide (GSSG) levels in *YAP1* overexpressing *P. pastoris* and controls. **Upper panel:** Intracellular GSH concentrations (µmol GSH per gram wet cell weight). Differences were not statistically significant (P-values > 0.1). **Middle panel:** Intracellular GSSG concentrations (µmol GSSG per gram wet cell weight). ** in the upper row indicate a significant difference to X-33 (P < 0.05). *** in the lower row indicates a significant difference to the trp strain (P < 0.01). **Lower panel:** Total glutathione (tGSH) concentration (intracellular: green (controls) or red (*YAP1* overexpressing strains), excreted: blue) was determined using the equation *t*GSH = [GSH] + 2*[GSSG].

As glutathione biosynthesis was among the up-regulated cellular processes in trpYAP1 (*GSH1*, *CYS3*), we investigated the levels of GSH and GSSG in the *YAP1* overexpressing strain and its parental control. No significant changes were determined for the concentration of reduced glutathione (Figure 2A). As shown previously 22, the strain secreting trypsinogen had a lower intracellular level of GSSG compared to the wild type strain X-33. Upon *YAP1* co-overexpression, GSSG levels in the trp strain are increased more than 50% and are even slightly higher than in X-33. This effect is specific for *YAP1* overexpression in the protein secreting strain, as GSSG levels were not increased in the wild type background (Figure 2B). On the contrary, GSSG levels are lower when overexpressing *YAP1* in the wild type background. Total glutathione content is slightly but not significantly lower in the engineered strains (Figure 2C). It seems, that enhanced Yap1 availability is able to restore the physiological redox conditions in the secreting strain to the wild type state. Again, GSH excretion does not seem to play a significant role, as extracellular GSH levels were almost identical in all strains (Figure 2C), while extracellular GSSG concentrations are below the limit of quantification (~0.007 µmol/g wet cell weight).

### Overexpression of the transcription factor Yap1 causes a slight but significant reduction of the ER redox state, but restores cytosolic redox state in the trypsinogen secreting strain to the wild type level 

**Figure 3:**
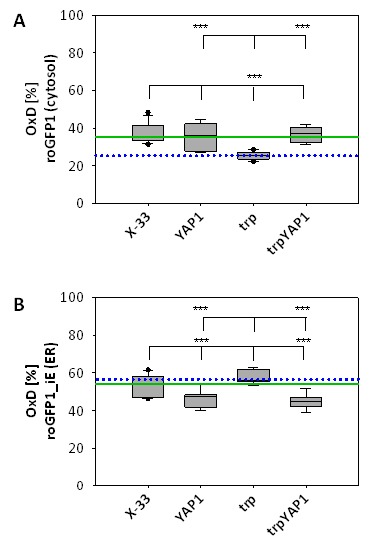
FIGURE 3: *YAP1* co-overexpression with trypsinogen reoxidizes the cytosol redox state to the level of the wild type strain, while slightly reducing the ER redox ratio. The OxD obtained with roGFP are represented as Box-and-Whisker plots. Each box is separated into two inter-quartiles by the statistical median as a horizontal line in the box. The extreme values extending from the inter-quartile at most 1.5 times from the upper or lower inter-quartile are the ‘whiskers’. The green line indicates the median of X-33, the blue dotted line represents the median of the trp strain. **(A) **Cytosolic redox ratios of the respective strains represented as OxD of roGFP. Statistical significance is indicated as for (B). **(B)** ER redox ratios of the respective strains represented as OxD of roGFP-iE. *** in the lower row indicate a significant difference to X-33 (P < 0.01). *** in the upper row indicates a significant difference to the trp strain.

As *YAP1*-overexpression seems to have a strong impact on intracellular redox conditions, we determined compartment-specific redox ratios in the cytosol and the ER using redox-sensitive GFP variants 27,41. We have already demonstrated that folding of proteins in the ER does not affect the redox environment of the ER, but leads to reduction of the redox state of the cytosol 22. Overexpression of *YAP1* in the strain producing the recombinant secretory protein reoxidizes the redox state of the cytosol to the level of the wild type strain (Figure 3A), which is also reflected by the increased levels of oxidized GSSG in the trpYAP1 strain. Again, the effect is specific for the recombinant protein secreting strain, which displayed the lower cytosolic redox state. Interestingly, *YAP1* overexpression had also an effect on the ER redox state, and caused a slight but significant reduction of the ER through a yet unidentified mechanism (Figure 3B).

### Conclusions

Oxidative protein folding in the ER generates stoichiometric amounts of ROS, in particular H_2_O_2_. At present, it is not clear whether these ROS stay within the ER or diffuse to the cytosol, but we could clearly show that Yap1 is involved in physiological detoxification of ROS formed upon oxidative folding in the ER. Yap1 is required to activate the antioxidant enzymes, which are quenching ROS. Cells with significantly lowered Yap1 levels react to increased secretory folding load with accumulation of ROS (e.g., hydrogen peroxide and superoxide anions) and strong flocculation. On the other hand, enhanced levels of Yap1 seem to create a more convenient environment for folding and secretion of (recombinant) proteins.

It should be noted that protein folding is not a stoichiometric process, but usually requires several rounds of folding per molecule. It has been reported previously, that per each disulfide bond formed one molecule of hydrogen peroxide is generated 9. Additionally, folding also requires ATP, as correct folding of nascent peptides by chaperones occurs along multiple ATP-driven cycles of substrate binding and release (recently reviewed e.g. by 42). Thus, increased mitochondrial activity to generate sufficient amounts of ATP might also contribute to ROS formation 43. At present we cannot distinguish between ER and mitochondria derived ROS. Based on the amount of secreted trypsinogen after 48 h in our study, at least 10^6^ molecules of trypsinogen were processed per cell in the trp strain, with each trypsinogen having six disulfide bonds to be correctly formed. Nevertheless, we did not observe ROS accumulation in this strain compared to the non-expressing wild type strain X-33, indicating that the cell’s antioxidant response can cope with this amount of secretory proteins. However, we did see ROS accumulation in strains with higher oxidative protein folding and secretion levels 22, indicating that the additional amount of secretory load overwhelms the antioxidant capacity of the cells.

We have previously shown that increased oxidative folding of proteins in the ER has a strong effect on the redox environment of the cytosol, leading to more reducing conditions 22. This cellular redox imbalance is stressful for the cells. Here we demonstrate that overexpression of *YAP1* in such a strain reverses the effect on the redox status within these two compartments. It causes re-oxidation of the cytosol to the level of the wild type strain, while slightly reducing the redox state of the ER. Constitutive co-overexpression of *YAP1* also leads to increased secretion levels of a recombinant protein. These alterations are also reflected by the levels of reduced and oxidized intracellular glutathione, whereby a significantly higher GSSG amount in the *YAP1* co-overexpressing strain compared to its parental strain was measured. Total glutathione levels were thereby not remarkably affected among the analysed strains. Thereby, the degree of oxidation (OxD = 2*[GSSG] / [tGSH]) of the whole cell was shifted from 10% in the wild type and the trp strain to 14% in the trp_YAP1 co-overexpressing strain. Taken together, these data indicate a strong impact of intracellular redox balance on the secretion of (recombinant) proteins without affecting protein folding *per se*.

This hypothesis is further supported by microarray data of the respective *P. pastoris* strains, as genes involved in the antioxidant response were the predominant group of genes regulated by overexpressed *YAP1, *whereas no effect on the transcription of folding related genes was observed.

With these findings we conclude that the re-establishment of suitable redox conditions by tuning the antioxidant capacity of the cell reduces cell stress caused by recombinant protein production, and thereby increases the secretion capacity of *P. pastoris*. Moreover, these findings are thought to have implications not only for improving biotechnological production processes, but also impact our understanding of the development of many aging related diseases, as both cases share the underlying molecular processes, which connect cellular redox conditions with folding related stresses in the ER.

## MATERIALS AND METHODS

### Strains and vectors 

The single copy trypsinogen expressing strain (trp) was described in 22. Briefly, this strain contains a single expression cassette of porcine trypsinogen under control of the P*_GAP_*(glyceraldehyde-3-phosphate-dehydrogenase) promoter using the *S. cerevisiae* alfa-mating factor pre-pro-leader for secretion and has been confirmed not to activate the UPR by qPCR previously. For overexpression of *P. pastoris YAP1, *the respective gene (PAS_chr4_0601) was amplified from X-33 genomic DNA, and cloned into a pPuzzle vector 44 under control of the P*_GAP_*promoter. The Zeocin resistance marker was flanked by loxP sites. The vector was integrated into the native *YAP1* locus of the *P. pastoris* genome after linearization in the respective sequence. After transformation by electroporation, positive transformants were selected on YPD plates with Zeocin.

For conditional downregulation of *YAP1, *the native P*_YAP1_* promoter was exchanged for the serine repressible P*_SER_* promoter using the strategy described in 28, resulting the trp∆*yap1*strain. The used primers are summarized in Table 2.

**Table 2:** Primers used for generation of *YAP1* overexpression and knock down.

**Primer name**	**Primer sequence**
Yap1_fw (*Sbf*I)	TAGACCTGCAGGATGAGTGACGTGGTAAACAAG
Yap1_rv (*Sfi*I)	AATAGGCCGAGGCGGCCCTATTTAAACATGGAAAAATCG
P*_SER1_*_fw (*Sbf*I)	TAGACCAAGGCCTTGG CAGCAAATAATTAGCAGCC
P*_SER1_*_rv (*BstX*I)	AATACCTGCAGG TGTATTATATGGTTAGTTCAAGATG
P*_YAP1_*_fw (*Asc*I)	ATTAGGCGCGCCGCAAAAGCGAGTACTATTTCTCAAA
P*_YAP1_*_rv (*Apa*I)	ATTAGGGCCCAGCGGAAGAACAACTTTAGTGAGTA

Vectors containing redox-sensitive GFP variants roGFP1 and roGFP1_iE for targeting the cytosol and the ER were described in 27 and were used for the transformation of the strains mentioned above.

For the localization of overproduced Yap1, superfolder GFP (sGFP) was fused to the C-terminus of the *YAP1* gene, and expressed under control of P*_GAP_*. These strains were then analysed by Western blot for expression and fluorescence microscopy for intracellular localization.

High level trypsinogen secreting strains were generated by overexpressing pTRP under control of P_GAP_ and selecting for the highest expressing *P. pastoris* strains containing multiple copies of the expression cassette on increased antibiotics (Zeocin) concentrations. For overexpression of *P. pastoris YAP1 *in these strains*, *the Zeocin resistance marker in the pPuzzle vector was exchanged for the KanMX cassette. The vector was integrated into the native *YAP1* locus of the *P. pastoris* genome after linearization in the respective sequence. After transformation by electroporation, positive transformants were selected on YPD plates with G418.

### Production of porcine trypsinogen

Screening media contained per liter: 10 g pea peptone, 10 g yeast extract, 10.2 g (NH)_2_HPO_4_, 1.24 g KCl, 910 µL 1M CaCl_2_ solution and 1 mL biotin stock solution (0.2 g/L). Pre-culture was performed in 5 mL YPD for all strains. For the main culture, 10 clones per construct were inoculated into 10 mL of screening medium in 100 mL shake flasks (at an OD_600_ of 0.1) and incubated at 28°C with 170 rpm (rotations per minute) for 48 h.

The amount of secreted trypsinogen was determined in the supernatant by measuring the activity of enterokinase activated trypsin according to the protocol established by 45. The sample buffer was exchanged to 1 mM HCl over PD-10 columns (Amersham Biosciences). Then, 50 µL of sample was incubated with 5 µg bovine enterokinase (Sigma) in 50 mM Tris/HCl buffer, pH 8.6, containing 50 mM CaCl_2_, to activate trypsinogen, and finally trypsin activity was determined with the TAME assay. Therefore, aliquots of the samples were added to 40 mM Tris/HCl buffer pH 8.1 buffer containing 10 mM CaCl_2_ and 1mM p-toluene sulfonyl-L-arginine ethylester-HCl (TAME), and the increase of absorbance at 247 nm was followed. Trypsinogen concentration is calculated using the extinction coefficient ϵ = 0.0101 E_247_ cm^-1^ min^-1^.

### Staining of reactive oxygen species (ROS)

The fluorescent probes DHE (dihydroethidium) and DHR (dihydrorhodamine 123) were used to determine levels of ROS as described in 22. Briefly, cells of an OD_600_ of 0.4 were harvested by centrifugation and resuspended in 2 mL PBS. DHE and DHR were added to cells at a final concentration of 10 μg/mL and incubated at 30°C for 30 min. Flow cytometry analysis was performed using a FACS Canto with the settings: DHR green filter (525-550 nm), DHE red filter (600-650 nm).

### Analysis of transcriptional regulation using DNA microarrays

The strains trp and trpYAP1 were cultivated in screening medium in 4 biological replicates each. 10 mL of main culture were inoculated from an overnight pre-culture with an OD_600_ of 4. After 6 h (exponential growth phase), samples were taken and fixed in phenol/ethanol, and stored at -80°C until total RNA extraction. Total RNA extraction, two-color labelling in a dye swap manner for each sample and cRNA hybridization to the *P. pastoris* specific microarrays were performed as in 34. Samples were hybridized to the microarrays described in Graf et al. (34; 8x15K custom arrays, AMAD-ID 018045, Agilent) and to a novel in house designed *P. pastoris* specific oligonucleotide array, which was based on the improved annotation of *P. pastoris* strain GS115 (8x15K custom arrays, AMAD-ID 034821, Agilent, 46). Raw data normalization was done using locally weighted MA-scatterplot smoothing (LOESS) followed by a between array ‘Aquantile` normalization, both available within the limma package of R 47. P-values were corrected for multiple testing using Benjamini & Yekutieli method 48. Features were defined as differentially expressed if they had a P-value <0.05. For the identification of stronger regulatory effects an additional cut-off for the fold change (FC) of ± 1.5 fold was applied. A high degree of correlation of significantly regulated genes between the two microarrays was determined (R^2^ = 0.902), thus the average FC of both microarrays was used.

### Determination of transcript levels by quantitative real time PCR

RNA isolation, cDNA synthesis and measurement of mRNA transcript levels using real time PCR was performed as described in 44 using the primers given in 22. The reference gene for normalization was actin (*ACT1*), each gene transcript was correlated to *ACT1* as internal control. The wild type strain X-33 served as reference strain for relative transcript level determination using the delta-delta C_t_ method.

### Cultivation conditions for redox and glutathione measurements

M2 minimal medium contained per liter: 20 g of glucose, 20 g of citric acid, 3.15 g of (NH_4_)_2_HPO_4_, 0.03 g of CaCl_2_ • 2H_2_O, 0.8 g of KCl, 0.5 g of MgSO_4_ • 7H_2_O, 2 mL of biotin (0.2 g L^-1^), 1.5 mL of trace salts stock solution. The pH was set to 5.0 with 5M KOH solution. Trace salts stock solution contained per liter: 6.0 g of CuSO_4_ • 5H_2_O, 0.08 g of NaI, 3.0 g of MnSO_4_ •H_2_O, 0.2 g of Na_2_MoO_4_ • 2H_2_O, 0.02 g of H_3_BO_3_, 0.5 g of CoCl_2_, 20.0 g of ZnCl_2_, 5.0 g of FeSO_4_ • 7H_2_O, and 5.0 mL of H_2_SO_4_ (95-98% w/w).

Pre-culture was performed in 5 mL M2 medium. 10 mL M2 medium in 100 mL shake flasks were inoculated at an OD600 of 0.5, and were incubated at 28°C with 170 rpm (rotations per minute) for 24 h. For each strain, 12 individual clones were analysed.

### Determination of redox ratios using roGFP

Redox states of all strains were measured during the exponential growth phase. Briefly, 840 µL culture were mixed with 60 µL 1.5 M K-MOPS buffer (pH 7.0). Total oxidation or reduction of the roGFPs was achieved by addition of 200 µL 6.3 mM 4,4′-dipyridyl disulfide (4-DPS) or 200 µL 1M dithiothreitol (DTT), respectively, and was compared to the untreated culture where 200 µL of PBS were added as control 49. The fluorescence of the cells was detected in 96-well plates (FluoroNunc, Nunc) on a fluorescence photometer (Infinite M200 Tecan plate reader), where it was possible to measure the excitation of two wavelengths, 395 and 465 nm, corresponding to the oxidized and the reduced form of the protein. Each sample was measured four times 27. The degree of oxidation (OxD) of the cytosol and the ER was calculated according to equation 1. The quotient of fluorescence intensities (equation 2) is the so called ‘instrument factor IF’ and corrects for variations in the signal strength from the instrument. R is the experimentally determined fluorescence ratio of the intensities for the two wavelengths 395 and 465 nm 50. R_ox_ and R_red_ stand for the ratios of fully oxidized or fully reduced roGFP, respectively, while R is the ratio of the untreated sample.

**Figure:**
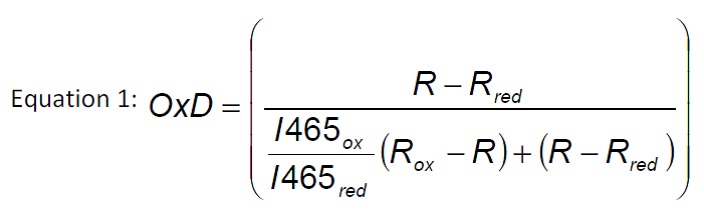


**Figure:**
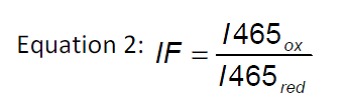


### Determination of glutathione concentrations by LC/MS-MS

Three pellets of 1 mL of exponential culture were collected by centrifugation at 3000 rpm for 5 min at 4°C. The cells were resuspended in 1 mL ice cold 0.1 M H_3_PO_4_, spiked with labeled glutathione-glycine-^13^C_2_,^15^N (Sigma Aldrich) and GSSG- glutathione-glycine-^13^C_2_,^15^N (synthesized in house) and heated to 75°C for 3 min to extract GSH and GSSG. The cell extracts were measured by LC/MS-MS (Agilent Ion Trap) using hydrophilic interaction chromatography (HILIC) and acetonitrile/water as eluent 51.

### Statistical analyses 

Parts of this work have been supported by the Federal Ministry of Science, Research and Economy (BMWFW), the Federal Minis-try of Traffic, Innovation and Technology (bmvit), the Styrian Business Promotion Agency SFG, the Standortagentur Tirol and ZIT - Technology Agency of the City of Vienna through the COMET-Funding Program managed by the Austrian Research Promotion Agency FFG. EQ BOKU VIBT GmbH is acknowledged for providing LC-MS/MS instrumentation. Finally, we thank the BOKU-VIBT Imaging Center for access and expertise with fluorescence microscopy equipment.

## SUPPLEMENTAL MATERIAL

Click here for supplemental data file.

All supplemental data for this article are also available online at http://microbialcell.com/researcharticles/overexpression-of-the-transcription-factor-yap1-modifies-intracellular-redox-conditions-and-enhances-recombinant-protein-secretion/.
